# Sense of coherence, social support and religiosity as resources for medical personnel during the COVID-19 pandemic: A web-based survey among 4324 health care workers within the German Network University Medicine

**DOI:** 10.1371/journal.pone.0255211

**Published:** 2021-07-26

**Authors:** Jonas Schmuck, Nina Hiebel, Milena Rabe, Juliane Schneider, Yesim Erim, Eva Morawa, Lucia Jerg-Bretzke, Petra Beschoner, Christian Albus, Julian Hannemann, Kerstin Weidner, Susann Steudte-Schmiedgen, Lukas Radbruch, Holger Brunsch, Franziska Geiser

**Affiliations:** 1 Department of Psychosomatic Medicine and Psychotherapy, Medical Faculty, University Hospital Bonn, Bonn, Germany; 2 Department of Psychosomatic Medicine and Psychotherapy, University Hospital of Erlangen, Friedrich-Alexander University Erlangen-Nürnberg, Erlangen, Germany; 3 Department of Psychosomatic Medicine and Psychotherapy, Ulm University Medical Center, University Ulm, Ulm, Germany; 4 Department of Psychosomatics and Psychotherapy, University Hospital Cologne, University Cologne, Cologne, Germany; 5 Department of Psychotherapy and Psychosomatic Medicine, Faculty of Medicine, Technische Universität Dresden, Dresden, Germany; 6 Department of Palliative Medicine, Medical Faculty, University Hospital Bonn, Bonn, Germany; Medical University of Vienna, AUSTRIA

## Abstract

**Introduction:**

The COVID-19 pandemic resulted in severe detrimental effects on the mental well-being of health care workers (HCW). Consequently, there has been a need to identify health-promoting resources in order to mitigate the psychological impact of the pandemic on HCW.

**Objective:**

Our objective was to investigate the association of sense of coherence (SOC), social support and religiosity with self-reported mental symptoms and increase of subjective burden during the COVID-19 pandemic in HCW.

**Methods:**

Our sample comprised 4324 HCW of four professions (physicians, nurses, medical technical assistants (MTA) and pastoral workers) who completed an online survey from 20 April to 5 July 2020. Health-promoting resources were assessed using the Sense of Coherence Scale Short Form (SOC-3), the ENRICHD Social Support Inventory (ESSI) and one item on religiosity derived from the Scale of Transpersonal Trust (TPV). Anxiety and depression symptoms were measured with the PHQ-2 and GAD-2. The increase of subjective burden due to the pandemic was assessed as the retrospective difference between burden during the pandemic and before the pandemic.

**Results:**

In multiple regressions, higher SOC was strongly associated with fewer anxiety and depression symptoms. Higher social support was also related to less severe mental symptoms, but with a smaller effect size, while religiosity showed minimal to no correlation with anxiety or depression. In professional group analysis, SOC was negatively associated with mental symptoms in all groups, while social support only correlated significantly with mental health outcomes in physicians and MTA. In the total sample and among subgroups, an increase of subjective burden was meaningfully associated only with a weaker SOC.

**Conclusion:**

Perceived social support and especially higher SOC appeared to be beneficial for mental health of HCW during the COVID-19 pandemic. However, the different importance of the resources in the respective occupations requires further research to identify possible reasons.

## Introduction

The first appearance of the coronavirus SARS-CoV-2 was reported in December 2019 in China [1]. Only three months later, the WHO declared the respiratory disease COVID-19 caused by SARS-CoV-2 a worldwide pandemic [2]. To slow the spread and reduce transmission of the virus, most countries went into lockdown in early 2020, resulting in severe restrictions on personal and professional life. While this was effective ‘in flattening the curve’ [3], it came at a great cost for mental well-being in the general population. Cross-sectional studies conducted in Germany in early 2020 showed that prevalence of mental health problems was significantly elevated among the general population [4]. Health care workers (HCW) in particular have been directly exposed to the hazards of the pandemic. Treating infected patients with sometimes inadequate protection, concerns about COVID-19 infection and working long hours with little sleep placed an enormous burden on the mental health of HCW [5, 6]. Research has shown that HCW involved in previous epidemics experienced high levels of anxiety and depression [7]. Similar findings have emerged during the first wave of the COVID-19 pandemic. A recent meta-analysis of 62 studies revealed that the pooled prevalence of anxiety and depression in HCW was 26% and 25%, respectively [8]. In Germany, in an online survey (VOICE study), Morawa et al. [9] found the prevalence across different groups of HCW ranging from 17.4% to 23.0% for depression symptoms and from 17.8% to 20.1% for anxiety symptoms. The data presented here originate from a subsample of this study.

While prevalence figures for mental health symptoms varied between different studies and countries, it was evident that HCW were exposed to high levels of psychological stress during the COVID-19 pandemic [10]. Since symptoms of acute stress, anxiety and depression can severely impair the well-being of HCW and their ability to work [11], it is crucial to identify resources that could protect against mental strain and disorders. Among the psychosocial concepts that have been highlighted in the literature are social support, religiosity/ spirituality and sense of coherence (SOC) [12–14].

Within the term social support, various concepts are typically summarized ranging from different types (e.g. emotional or instrumental support) to direction (e.g. uni- or bidirectional) of support [15]. One concept which is considered highly beneficial for people’s well-being is perceived social support (i.e., perceptions of availability of high-quality support). It has repeatedly been negatively associated with mental health problems such as depression and distress [13]. During the COVID-19 pandemic, perceived social support turned out to be protective against depression [16] and psychological distress [17] in the general population. These findings have been confirmed in studies with HCW. Both anxiety [18] and stress levels [19] during the pandemic were significantly reduced by greater perceived social support. An analysis of another subsample of HCW from the current survey showed that social support had stronger associations with depression and anxiety than demographic or occupational risk factors [20].

SOC is one of the key resilience concepts in the theory of salutogenesis proposed by Antonovsky [21]. It is a global orientation that reflects the degree to which people perceive their world as comprehensible, manageable and meaningful. Reviewing 458 studies, Eriksson and Lindström [12] found SOC to be a major predictor for mental health in the general population, as a strong SOC was negatively related to depression, anxiety and post-traumatic stress disorder. In health care settings, higher SOC was linked to fewer mental health problems among nurses [22], paramedics [23] and intensive care unit staff [24]. Similar results were recently established in a sample of Spanish HCW during the COVID-19 pandemic [25].

It has been suggested that religiosity and the broader concept of spirituality may also play an important role in mental well-being. Previous research indicated a stress-reducing effect of religiosity [26] and a positive association between religiosity and mental health [27]. However, findings regarding the influence of religiosity on mental health during the COVID-19 pandemic have been inconclusive. While a protective effect of religiosity on COVID-19-related worries was found by Kranz et al. [28], others reported that religious people had higher levels of fear [29] or showed no difference compared to non-religious people [30]. Among American nurses, high spirituality was associated with less depression and anxiety [31]. To our knowledge, results on the role of religiosity in HCW in the COVID-19 pandemic in Germany have not been published to date.

To sum up, although individual health-promoting resources during the COVID-19 pandemic have been investigated before, various internal and external resources have not yet been examined simultaneously in German HCW. In addition, little is known about the resources of different medical professional groups during the COVID-19 pandemic. Most research in the health sector so far has focused only on physicians and nurses. Due to their importance in the COVID-19 pandemic, we included MTA [9] and pastoral workers [32] as additional groups. The main objective of the study was to investigate the association of social support, SOC and religiosity on mental health and increase of subjective burden due to the COVID-19 pandemic in a large German sample of HCW taking occupational and workplace differences into account. In line with previous findings, the first hypothesis was that higher levels of social support, SOC and religiosity were associated with lower levels of depression and general anxiety. Secondly, it was hypothesized that all three resources were associated with a smaller increase in burden levels caused by the COVID-19 pandemic. The results of the present study will be discussed within the newly set-up German cooperation network of university medicine by the German Federal Ministry of Education and Research (BMBF). The analyses of our survey are intended to point the way forward for the planning of future protective measures.

## Materials and methods

### Data collection

The VOICE online survey was conducted between April 20 and July 5, 2020, with the aim to reach as many health care professionals as possible in the defined time period. The psychosomatic departments of the university hospitals of Bonn, Erlangen, Ulm, Cologne, and Dresden, Germany, provided the link through online platforms or mailing lists for the staff of their university hospitals. Participation in the survey was encouraged by numerous other general hospitals as well as several professional associations and professional online platforms. The background for the study presented here was a research project on resilience in religion and spirituality; data evaluation of the VOICE survey was performed as part of the collaborative research project egePan Unimed within the newly set-up German Network University Medicine. The aim of egePan Unimed is to examine and coordinate management concepts of the pandemic in Germany and internationally, to evaluate their practicability using scientific methods and to manage them within a framework plan. Superior aims include an adequate control of resources within a region in order to avoid an inefficient occupancy and intensive care supply in an inpatient setting and a case management for both hospitalized and non-hospitalized patients. The present study was approved by the Ethics Committee of the Medical Faculty of the Rheinische Friedrich Wilhem University Bonn (reference number: 125_20) and Medical Faculty of the Friedrich-Alexander University Erlangen-Nürnberg (FAU) (reference number: 133_20 B) and registered on ClinicalTrials (DRKS-ID: DRKS00021268). All respondents provided their online informed consent.

The 15-minute survey in German language could be accessed via two academic online survey tools, Unipark (www.unipark.com) and SoSci Survey (www.soscisurvey.de). The survey was made up of 77 items. Inclusion criteria were a minimum age of 18 years, working in the health care sector, residence/working place in Germany, and sufficient German language skills. We only included participants in our analysis with complete data sets who worked as a physician, nurse, medical technical assistant (MTA) or pastoral worker in the inpatient sector (university hospital and other hospital) or the outpatient sector (doctor’s practice and medical care centre). The term MTA refers to allied medical staff such as laboratory or radiology or pharmaceutical-technical assistants who have undergone three years of professional training.

### Sample characteristics

A convenience sample of 8071 health care professionals took part in the online survey. Of these, 1291 worked in a social pediatric center who, for reasons of representativity, were not included in the analysis. Furthermore, 2456 participants who had missing data or could not clearly be allocated to one of the four medical professions (physician, nurse, MTA, pastoral worker) or to either the inpatient or the outpatient sector were also excluded. 4324 participants remained in the sample. The majority worked in the inpatient sector (*n* = 3496, 80.9%) rather than in the outpatient sector (*n* = 828, 19.1%). Most of the participants were employees of four university hospitals, Cologne (*n* = 565, 13.1%), Bonn (*n* = 490, 11.3%), Erlangen (*n* = 461, 10.7%) and Dresden (*n* = 266, 6.2%). Regarding the four different medical profession groups, 34.5% (*n* = 1492) of the total sample (*N* = 4324) reported to work as a physician, 27.1% (*n* = 1171) as a nurse, 34.9% (*n* = 1509) as MTA and 3.5% (*n* = 152) as pastoral worker. We cannot report response rates for the total sample due to the heterogeneous recruitment strategy; however, we can report the response rates for the four university hospitals with the largest proportions of respondents, which were study centers or cooperation partners. The highest average response rate was found for the MTA (22.5%, range: 13.2–33.8%), followed by physicians (9.4%, range: 7.6–12.7%), and nurses (8.1%, range: 5.2–9.4%). The mean gender proportion (females to males) for the three groups was 59.7%:40.3% (respondents) and 49.0%:51.0% (hospitals) for physicians; 75.6%:24.4% (respondents) and 77.9%:22.1% (hospitals) for nurses, and 82.7%:17.3% (respondents) and 92.7%:7.3% (hospitals) for MTA. The response rate for pastoral workers could not be measured as we had no knowledge about the total number of pastoral workers working at the hospitals at the time of publication. Over 90 percent of the HCW participated within the first month after the study was launched (April 20 - May 19).

### Measures

#### Mental health and subjective burden level

Mental health symptoms were assessed with the PHQ-4 (Patient Health Questionnaire) [33]. This ultrashort form (4 items) of the Patient Health Questionnaire (PHQ-D) is divided into two separate modules (PHQ-2 and GAD-2). The PHQ-2 measures depression levels whereas the GAD-2 assesses generalized anxiety, both with two items ranging from 0 („not at all“) to 3 („nearly every day“). For example, in the first PHQ-2 item, participants were asked to report how often they “felt down, depressed, or hopeless” over the last two weeks. The first GAD-2 item asked how often the participants “felt nervous, anxious or on edge” over the last two weeks. The aggregate sum score for each module ranges from 0 to 6. A cut-off value from ≥3 for each module has been suggested to identify likely cases of depression or anxiety. The psychometric characteristics of the scales are well established [33]. In the present sample, the validated German version received acceptable Cronbach’s alpha scores of 0.76 for PHQ-2 and 0.78 for GAD-2.

The overall burden level during and (retrospectively) before the COVID-19 pandemic was assessed on a single item basis. Participants were asked “How much burden have you felt due to the COVID-19 pandemic in the last 2 weeks?” and “How much burden did you feel before the COVID-19 pandemic?”, respectively. The Likert-type scale ranged from 0 "not at all" to 4 "very strong". To determine the increase in subjective burden levels we calculated a difference score between these two items (during the pandemic–before the pandemic). Higher scores should therefore reflect a higher increase in subjective burden due to the COVID-19 pandemic.

#### Sense of coherence

SOC is a substantial personal resource for well-being and refers to the person’s view of the world as coherent. In the present study, SOC was assessed using a German ultrashort version (SOC-3) [34] of the original SOC scale developed by Antonovsky [35]. The SOC-3 is a highly economic instrument with sufficient reliability that shows strong correlations with the original SOC scale [34]. Only two of the three subscales from the original version are present in the SOC-3, namely comprehensibility and meaningfulness. It measures SOC based on three items. Two of them are rated on a scale ranging from 1 (“very often”) to 7 (“very seldom or never). An example item is “Do you have very mixed-up feelings and ideas?”. The third item (“When you think about your life, you very often…) is rated from one (“feel how good it is to be alive”) to seven (“ask yourself why you exist at all”) and has to be inverted. The final SOC-3 sum score therefore results in a range from 3 to 21. Higher values in the SOC-3 indicate a stronger SOC. In the present sample, Cronbach’s alpha was 0.71.

#### Social support

Social support was measured using the German version of the ENRICHD Social Support Inventory (ESSI-D) [36]. The original scale was developed for people who had suffered a myocardial infarction [37] but has also been recommended as a valid and highly reliable measurement for perceived social support. It focuses particularly on emotional social support by close friends or family. The ESSI-D comprises five items which are rated on a scale from one (”never”) to five (”always”). An example item is “If you need a conversation, is there someone who listens to you properly?“. The final ESSI score is obtained by adding all five item scores together, which results in a range from 5 to 25. Higher scores indicate higher levels of perceived social support. Low social support is defined as scale value of ≤ 18 and the answer of at least two items ≤ 3 [36]. In the present sample, the ESSI-D obtained a good Cronbach’s alpha score of 0.89.

#### Religiosity

To measure the participants’ degree of religiosity, one item derived from the scale of Transpersonal Trust (TPV-11) [38] was used. The TPV-11 and this particular item assess a person’s connection with a higher being and therefore relate to aspects of spirituality and religiosity. The following item (here in own translation) was presented to participants: “I feel connected with a higher reality/ with a higher being/ with God. Even in hard times I can trust on this.” The item was rated on a four-point scale ranging from 0 “is not true at all” to 3 “is completely true”.

#### Sociodemographic, occupational, and COVID-19-related variables

The online questionnaire consisted of several sociodemographic, occupational and COVID-19-related characteristics. The following data were used in our study: age group, gender, profession, work setting, years of professional experience and having direct contact at work with COVID-19 infected patients proved by a test or having contact with contaminated material during work. Having contact with either COVID-19 infected patients or contaminated material was aggregated into the following variable: Having contact with SARS-CoV-2. Response categories for all items are presented in Table 1. Additionally, the week of participation for each participant was also recorded.

**Table 1:** Descriptive statistics for sociodemographic and occupational variables for the different professions.

	Physicians (*n* = 1492)	Nurses (*n* = 1171)	MTA (*n* = 1509)	Pastoral workers (*n* = 152)	Total sample (*N* = 4324)
**Gender, *n* (%)**					
male	603 (40.4)	277 (23.7)	188 (12.5)	64 (42.1)	1132 (26.2)
female	889 (56.6)	894 (76.3)	1321 (87.5)	88 (57.9)	3192 (73.8)
**Age, years, *n* (%)**					
18–30	143 (9.6)	319 (27.2)	318 (21.1)	0	780 (18.0)
31–40	390 (26.1)	294 (25.1)	339 (22.5)	3 (2.0)	1026 (23.7)
41–50	356 (23.9)	252 (21.5)	345 (22.9)	19 (12.5)	972 (22.5)
51–60	428 (28.7)	259 (22.1)	434 (28.8)	95 (62.5)	1216 (28.1)
>60	175 (11.7)	47 (4.0)	73 (4.8)	35 (23.0)	330 (7.6)
**Contact with SARS- CoV-2, *n* (%)**^**a**^					
yes	840 (56.3)	684 (58.4)	1141 (75.6)	75 (49.3)	2740 (63.4)
no	652 (43.7)	487 (41.6)	368 (24.4)	77 (50.7)	1584 (36.6)
**Professional experience, *n* (%)**					
<3 years	132 (8.8)	76 (6.5)	96 (6.4)	24 (15.8)	328 (7.6)
3–6 years	143 (9.6)	161 (13.7)	123 (8.2)	19 (12.5)	446 (10.3)
>6 years	1165 (78.1)	907 (77.5)	911 (60.4)	81 (53.3)	3064 (70.9)
unknown	52 (3.5)	27 (2.3)	379 (25.1)	28 (18.4)	486 (11.2)
**Work setting, *n* (%)**					
Inpatient	992 (66.5)	1159 (99.0)	1193 (79.1)	152 (100)	3496 (80.9)
outpatient	500 (33.5)	12 (1.0)	316 (20.9)	0 (0)	828 (19.1)

*Note*. ^a^ Having contact with either COVID-19 infected patients or contaminated material.

The survey also included further sociodemographic, occupational and COVID-19-related variables as well as questionnaires measuring working conditions and potential problems in the COVID-19 pandemic, posttraumatic stress disorder symptoms, work family conflict, and effort and reward imbalance at work. The results for these questionnaires have been [9, 20] or will be analysed in other publications.

### Statistical analysis

All data analyses were conducted with SPSS V. 27. Descriptive statistics (absolute and relative frequencies for categorical variables or mean and standard deviation for continuous variables, respectively) were calculated to describe the demographic and work-related characteristics, health-promoting factors (SOC, social support and religiosity), mental health (anxiety and depression) and increase in burden for each profession separately and in the whole sample. Group differences regarding the health-promoting resources, anxiety and depression symptoms and increase in subjective burden were tested with the univariate analysis of variance (Welch’s ANOVA) [39]. If the omnibus test was significant, Welch’s *t*-tests with Bonferroni adjusted *p-*values were conducted. The effect size (η²_p_) was also reported (η^2^_p_ ≥ 0.01 = small, η^2^_p_ ≥ 0.06 = medium and η^2^_p_ ≥ 0.14 = large effect size) [40]. To explore relationships between all variables of interest a correlation matrix was computed. Pearson’s correlation coefficients and, in the case of ordinal variables, Spearman`s correlation coefficients were reported. Multiple linear regression analyses were used to evaluate the influence of the potential resources on mental health and increase in subjective burden. Especially in large data sets, the performance of linear regression has been shown to be robust [41]. A hierarchical multiple linear regression model with SOC, social support, religiosity and control variables (age, gender, professional experience and contact with SARS-CoV-2) as predictors was calculated for each dependent variable (PHQ-4, PHQ-2, GAD-2, increase in burden) for the total sample and stratified for profession and work setting (outpatient or inpatient). Therefore, independent variables were entered in two steps into the model. To account for confounding factors, control variables were entered first. In a second step, the three resources were entered simultaneously into the regression model. A visual inspection of the residual plots confirmed a linear relationship between residuals and predicted values. The influence of each variable was assessed by using the standardized β-coefficient and its *p*-value. Since the control variables were not the focus of the analysis, only the coefficients and test statistics for the control variables and resources from the second step (except for *R*²) are presented in the tables. A negative β-coefficient should indicate a protective effect of the respective resource on mental health and increase in burden. Since we focused on the PHQ-2 and GAD-2, the regression for the PHQ-4 sum score is reported in Table 1 in S1 File. We also tested for effects related to the time of participation. Since there were no influences of the week of participation in any linear regression model we reported the results as presented above. A level of significance of *p* < .05 (two-tailed) was predetermined in all analyses except for the case of alpha error correction (then explicitly reported in the text).

## Results

### Sociodemographic and occupational characteristics

The descriptive statistics for sociodemographic and occupational variables are shown in Table 1 for each profession separately and for the total sample. Nearly three quarters of the participants (73.8%) were female with the physicians group having the lowest proportion of women (56.6%) and the MTA group the highest proportion (87.5%). The median age of the whole sample was between 41–50 years. Nurses tended to have a younger age on average while physicians and pastoral workers in particular were represented to a greater extent in the older age groups. The majority of participants (70.3%) had more than six years of professional experience, the group of physicians reporting the highest proportion of long work experience (78.1%) and the pastoral workers the lowest proportion (53.3%). Nearly two thirds of all participants (63.4%) indicated that they had contact with the SARS-CoV-2 virus, either through infected patients or contaminated material. While only half of the pastoral workers reported coming in contact with the virus (49.3%), over three quarters of the MTA did (75.6%).

### Health-promoting resources in HCW

Descriptive statistics for the measures of health-promoting factors and mental health as well as comparisons with reference values are presented in Table 2. The average value for SOC (SOC-3) was 15.39 (*SD* = 3.77), for social support (ESSI-D) 20.65 (*SD* = 3.98) and for religiosity (TPV) 1.14 (*SD* = 1.07). According to the cut-off criteria (value of ≤ 18 and the answer of at least two items ≤ 3), 24% of the total sample indicated a low level of social support. To determine differences between the professions, an ANOVA was performed for each resource measured. Results indicated that there was a significant effect of profession on SOC (*F*(3, 720.21) = 35.95; *p* < .001, η^2^_p_ = .02), on social support (*F*(3, 699.41) = 8.64, *p* < .001, η^2^_p_ = .01) and on religiosity (*F*(3, 781.00) = 370.91, *p* < .001, η^2^_p_ = .09). Pairwise comparisons with adjusted alpha error levels (Table 2 in S1 File) showed that pastoral workers had a significantly stronger SOC and MTA had a significantly weaker SOC than physicians and nurses. Levels of social support were higher in physicians and nurses than in MTA. Pastoral workers did not differ significantly from the three professions. Pastoral workers scored highest on religiosity, while physicians still reported higher levels than nurses and MTA.

**Table 2:** Mean scores and standard deviations for resources, mental health and increase in burden.

	Physicians (*n* = 1492)	Nurses (*n* = 1171)	MTA (*n* = 1509)	Pastoral workers (*n* = 152)	Total sample (*N* = 4324)
*Resources*					
**Sense of Coherence (SOC-3)**	15.72^a^ (3.63)	15.68^a^ (3.69)	14.69^b^ (3.92)	16.91^c^ (2.93)	15.39^1^ (3.77)
**Social Support (ESSI-D)**	20.79^a^ (3.78)	20.97^a^ (3.94)	20.23^b^ (4.19)	21.02^ab^ (3.78)	20.65^2^ (3.98)
**Religiosity (TPV)**	1.23^a^ (1.05)	1.02^b^ (1.05)	.99^b^ (1.00)	2.70^c^ (.58)	1.14 (1.07)
*Mental health*					
**Depression (PHQ-2)**	1.50^a^ (1.40)	1.69^b^ (1.48)	1.89^c^ (1.53)	1.20^a^ (1.05)	1.68 (1.47)
**Anxiety (GAD-2)**	1.52^a^ (1.50)	1.47^ac^ (1.53)	1.71^b^ (1.60)	1.13^c^ (1.08)	1.56 (1.54)
*COVID-19 burden*					
**Increase in burden**	.56^a^ (1.29)	.54^a^ (1.23)	.63^a^ (1.21)	.57^a^ (1.07)	.58 (1.24)

*Notes*. ^a-c^ Significant mean differences according to Bonferroni adjusted *t-*tests. Same letters indicate no significant mean differences between the respective professional groups.

^1^ The value is lower than in the German population (*M* = 16.03, *SD =* 3.44, *t*(4462) = 6.76, *p* < .001, *Cohen’s d* = .18) [34].

^2^ Not statistically different from the German population (*M* = 20.47, *SD =* 4.05, *t*(5276) = 1.79, *p* = .073) [42].

### Mental health and increase in burden in HCW

In relation to mental health symptoms, both anxiety (*F*(3, 735.38) = 13.83; *p* < .001, η^2^_p_ = .01) and depression scores (*F*(3, 732.08) = 28.35; *p* < .001, η^2^_p_ = .02) differed between occupations. The average score for depression symptoms (PHQ-2) was 1.68 (*SD* = 1.47) and for anxiety symptoms (GAD-2) 1.56 (*SD* = 1.54). The proportion of probable cases of a clinical depression (scores ≥ 3) or generalized anxiety in the total sample was 22% and 21%, respectively. The pairwise comparisons with adjusted alpha error levels (Table 2 in S1 File) indicated that MTA had the highest scores for depression levels and physicians and pastoral workers the lowest. Nurses reported intermediate levels. Anxiety levels were highest in MTA while pastoral workers scored lower than physicians. Nurses’ scores did not differ significantly from the latter groups. Levels of subjective burden during the COVID-19 pandemic were slightly increased in comparison to reported burden levels before the beginning of the pandemic (*M* = .58, *SD* = 1.24). However, there were no differences in the increase between the four professional groups (*F*(3, 709.92) = 1.58; *p* = .193, η^2^_p_ = .00).

### Resources associated with mental health and increase in burden

Table 3 illustrates the bivariate correlations among all variables that are included in the regression models. Results revealed that female gender in particular showed a small, but significant positive association with social support, anxiety and depression scores and a negative association with SOC. Older age was positively related to SOC and religiosity and negatively related to social support and depression. There was evidence of a robust relationship between the resources and mental health. While the link was most pronounced for SOC, social support demonstrated medium negative correlations with mental health symptoms as well. Associations with religiosity were comparatively weak.

**Table 3:** Correlations for demographics, resources, mental health symptoms and increase in burden.

Variable	1	2	3	4	5	6	7	8	9
1. Gender^a^	—								
2. Age^b^	-.07**	—							
3. Contact with SARS-CoV-2^c^	-.00	-.10**	—						
4. Sense of Coherence	-.08***	.18***	-.04**	—					
5. Social Support	.04**	-.11***	-.03	.39***	—				
6. Religiosity	.01	.21***	-.03*	.10***	.05***	—			
7. Depression	.06***	-.10***	.02	-.58***	-.29***	-.09***	—		
8. Anxiety	.09***	-.01	.05**	-.61***	-.29***	-.03	.66***	—	
9. Increase in burden	.05**	.08***	.01	-.13***	-.04*	.05**	.19***	.23***	—

*Notes*. *N* = 4324. ^a^ 0 = male and 1 = female.

^b^ Spearman’s Rho Coefficient is reported for categorical variable Age.

^c^ Having contact with either COVID-19 infected patients or contaminated material; 0 = no and 1 = yes.

*** *p* < .001.

** *p* < .01.

* *p* < .05.

To examine the influence of the resources on anxiety and depressive symptoms and the increase of subjective burden, we performed multiple linear regression analyses for the total sample, for each of the four professions and for the outpatient and inpatient sector. All models included age, gender, professional experience and contact with SARS-CoV-2 as control variables and SOC, social support and religiosity as predictors of interest.

**Fig 1:**
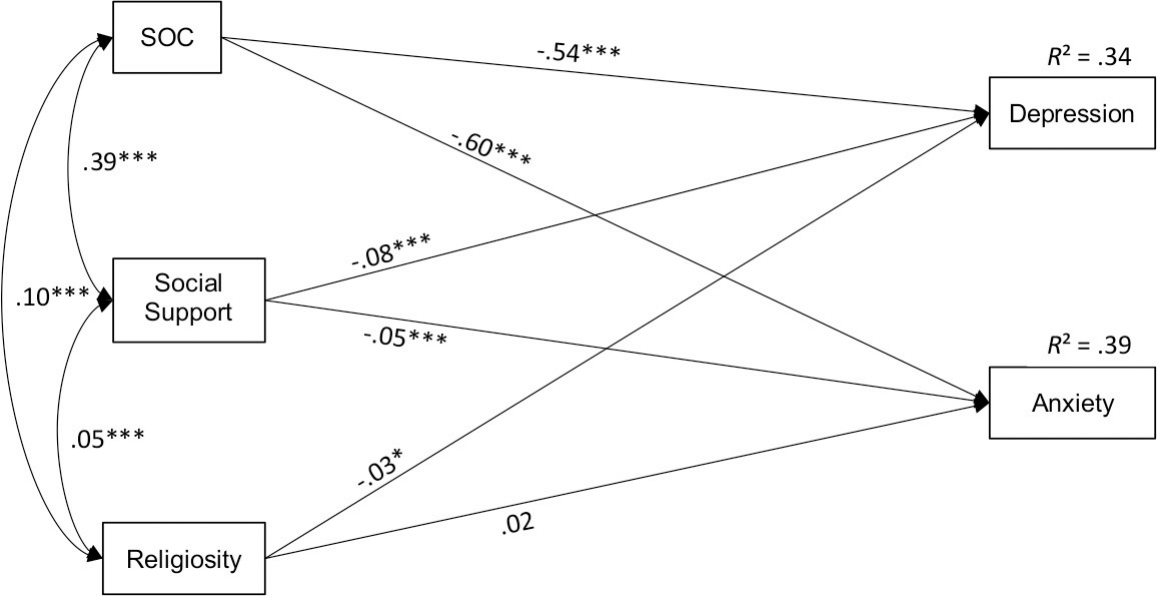
Path diagram to visualize the results of the regression analyses for the total sample. Shown are standard regression weights above the arrows, Pearson’s correlation coefficients between the predictors and adjusted squared multiple correlations for each dependent variable above its box. Control variables included Gender, Age, Professional Experience and Contact with SARS-CoV-2. *** p < .001. ** p < .01. * p < .05.

The regression models for depressive and anxiety symptoms in the total sample are provided in Table 4 and the results are summarized as a path diagram in Fig 1. While symptoms of depression were predicted by SOC (β = -.541, *p* < .001), social support (β = -.082, *p* < .001) and religiosity (β = -.031, *p* = .016), only SOC (β = -.601, *p* < .001) and social support (β = -.050, *p* < .001) displayed a relationship with symptoms of anxiety. Both models explained just over one third of the variance in the outcome variables. When the regression analyses were performed separately for the four professions (Tables 3–6 in S1 File), the following associations were found to be significant. For physicians, SOC (β = -.556, *p* < .001) and social support (β = -.095, *p* < .001) were significantly associated with lower severity of depressive symptoms. SOC (β = -.578, *p* < .001) and social support (β = -.074, *p* < .001) predicted a lower severity of anxiety symptoms as well. For nurses, only SOC and not social support was negatively associated with symptoms of depression (β = -.548, *p* < .001) and anxiety (β = -.605, *p* < .001). MTA with a stronger SOC (β = -.528, *p* < .001) and higher social support (β = -.098, *p* < .001) reported less symptoms of depression. In addition, SOC (β = -.619, *p* < .001) and social support (β = -.075, *p* < .001) were protective against anxiety symptoms in the MTA group. Among pastoral workers, a stronger SOC was related to less severity of depressive symptoms (β = -.385, *p* < .001) and anxiety symptoms (β = -.415, *p* < .001), but social support did not reach a significant level of prediction. Across all occupational groups, religiosity was not a significant predictor for anxiety and depression symptoms.

**Table 4:** Regression analyses for depressive (PHQ-2) and anxiety symptoms (GAD-2).

Independent Variables	PHQ-2 (*F*(12, 4311) = 187.092, *p* < .001, adjusted *R*² = .341)						
	*R²*	Δ *R²*	B (95% CI)	SE	β	*t*	*p*
**Step 1**: Control Variables	.015	.015					
Gender^a^			.032 [-.050, .114]	.042	.010	.766	.444
Age^b^							
31–40			-.110 [-.238, .018]	.065	-.032	-1.681	.093
41–50			-.177 [-.316, -.039]	.070	-.050	-2.517	**.012**
51–60			-.088 [-.223, .048]	.069	-.027	-1.266	.206
>60			-.223 [-.401, -.044]	.091	-.040	-2.433	**.015**
Professional experience^c^							
3–6 years			.159 [-.012, .330]	.087	.033	1.824	.068
>6 years			.250 [.090, .409]	.081	.077	3.074	**.002**
unknown			.292 [.110, .475]	.093	.063	3.143	**.002**
Contact with SARS-CoV-2^d^			-.034 [-.108, .040]	.038	-.011	-.900	.368
**Step 2**: Resources	.342	.327					
Sense of Coherence			-.211 [-.221, -.200]	.005	-.541	-39.050	**< .001**
Social Support			-.030 [-.040, -.020]	.005	-.082	-5.980	**< .001**
Religiosity			-.042 [-.076, -.008]	.017	-.031	-2.415	**.016**
	GAD-2 (*F*(12, 4311) = 228.150, *p* < .001, adjusted *R*² = .387)						
	*R²*	Δ *R²*	B (95% CI)	SE	β	*t*	*p*
**Step 1**: Control Variables	.015	.015					
Gender^a^			.178 [.095, .261]	.042	.051	4.211	**< .001**
Age^b^							
31–40			.112 [-.017, .241]	.066	.031	1.702	.089
41–50			.225 [.086, .364]	.071	.061	3.166	**.002**
51–60			.361 [.224, .498]	.070	.106	5.163	**< .001**
>60			.183 [.003, .363]	.092	.032	1.991	**.047**
Professional experience^c^							
3–6 years			-.131 [-.304, .041]	.088	-.026	-1.492	.136
>6 years			.087 [-.073, .248]	.082	.026	1.067	.286
unknown			-.008 [-.192, .176]	.094	-.002	-.083	.934
Contact with SARS-CoV-2^d^			.090 [.016, .165]	.038	.028	2.368	**.018**
**Step 2**: Resources	.388	.374					
Sense of Coherence			-.245 [-.256, -.234]	.005	-.601	-45.022	**< .001**
Social Support			-.019 [-.029, -.009]	.005	-.050	-3.774	**< .001**
Religiosity			.027 [-.008, .061]	.018	.018	1.509	.131

*Notes*. *N* = 4324. Δ *R²* = Change in *R²*. SE = Standard Error.

^a^ Reference group = male.

^b^ Reference group = Age 18–30.

^c^ Reference group = Professional experience <3 years.

^d^ Having contact with either COVID-19 infected patients or contaminated material; Reference group = no.

Examining the data separately for each work setting (Tables 7, 8 in S1 File) revealed that for HCW in the inpatient sector, SOC (β = -.531, *p* < .001), social support (β = -.089, *p* < .001) and religiosity (β = -.031, *p* = .029) were significant predictors of less severe depressive symptoms. However, only SOC (β = -.591, *p* < .001) and social support (β = -.049, *p* = .001) were predictive for anxiety symptoms. In the outpatient sector, SOC was the most important factor associated with decreased severity of depressive (β = -.586, *p* < .001) and anxiety symptoms (β = -.635, *p* < .001). Social support was negatively related to depressive symptoms (β = -.060, *p* = .049) but not to anxiety symptoms.

In terms of the increase in subjective burden in comparison to the time before the pandemic, all three predictors appeared to be significantly associated with the dependent variable in the total sample (Table 5). An increase in burden was associated with less SOC (β = -.166, *p* < .001). Increase in burden showed a statistically significant, but very small association with more social support (β = .037, *p* = .026) and stronger religiosity (β = .043, *p* = .005). Less than 4% of the variance were explained. However, when analysing all professional groups and work settings separately (Tables 9–11 in S1 File), a strong SOC remained the best predictor for less subjective increase in burden in all analyses except for pastoral workers (coefficients ranging from β = -.124 to β = -.225). High levels of religiosity predicted a minimal increase in burden only for physicians (β = .057, *p* = .025) and HCW in the inpatient sector (β = .051, *p* = .003) while social support had no significant associations with increase in burden at all. The amount of explained variance was relatively low in all analyses (no more than 6.8%).

**Table 5:** Linear regression analysis for subjective increase in burden for the total sample.

Independent Variables			Model 3: Increase in Burden (*F*(12, 4311) = 15.025, *p* < .001, adjusted *R*² = .037)				
	*R²*	Δ *R²*	B (95% CI)	SE	β	*t*	*p*
**Step 1**: Control Variables	.016	.016					
Gender^a^			.113 [.028, .197]	.043	.040	2.623	**.009**
Age^b^							
31–40			-.077 [-.208, .053]	.067	-.027	-1.160	.246
41–50			.156 [.015, .297]	.072	.052	2.165	**.030**
51–60			.146 [.007, .285]	.071	.053	2.066	.**039**
>60			.290 [.107, .472]	.093	.062	3.111	**.002**
Professional experience^c^							
3–6 years			.122 [-.053, .297]	.089	.030	1.371	.171
>6 years			.314 [.151, .477]	.083	.115	3.783	**< .001**
unknown			.278 [.091, .464]	.095	.071	2.920	**.004**
Contact with SARS-CoV-2^d^			.052 [-.024, .128]	.039	.020	1.348	.178
**Step 2**: Resources	.040	.024					
Sense of Coherence			-.055 [-.066, -.044]	.006	-.166	-9.945	**< .001**
Social Support			.011 [.001, .022]	.005	.037	2.221	**.026**
Religiosity			.050 [.015, .0.85]	.018	.043	2.819	**.005**

*Notes*. *N* = 4324. Δ *R²* = Change in *R²*. SE = Standard Error.

^a^ Reference group = male.

^b^ Reference group = Age 18–30.

^c^ Reference group = Professional experience <3 years.

^d^ Having contact with either COVID-19 infected patients or contaminated material; Reference group = no.

## Discussion

We provide results of a large cross-sectional study on psychosocial resources and mental health in HCW during the COVID-19 pandemic in Germany. In regression models, a strong SOC emerged as the strongest predictor for less severe symptoms of anxiety and depression among HCW. The results also supported negative, albeit weaker, associations of social support with anxiety and depression while religiosity had a marginal relation only with depressive symptoms. Furthermore, SOC appeared to be the only resource that contributed substantially to a lower increase in subjective burden with social support and religiosity showing effects that were too small to allow meaningful interpretations. This study underlines the importance of social support, and especially SOC, as potential resources for the mental health of HCW and strengthen previous findings from other countries during the COVID-19 pandemic [18, 19, 25].

Few studies have used the SOC-3 and the ESSI-D scales so far which limits the ability to interpret the obtained values in this survey. Comparisons with representative samples of the pre-pandemic German population [34] revealed that SOC was significantly weaker in the present sample, though the effect size was small. Even though SOC is conceptualized as a stable disposition, recent research showed that SOC may be reduced in individuals with high stress levels [43] which likely applies to HCW during the pandemic. Interestingly, levels of perceived social support did not differ from findings before the pandemic [36] even though restrictions had been imposed on social life during the pandemic. One explanation may be that among HCW who kept working throughout the pandemic, social support in the working team supposedly did not decrease and family support was still present. Support from significant others outside the family might still have been perceived as existing, even when less or only digitally accessible. Levels of religiosity could not be compared with other studies. In terms of mental symptoms, nearly every fifth HCW reported clinically relevant symptoms of depression and anxiety. This is a higher proportion than in COVID-19 studies that used the PHQ-2 and GAD-2 among Chinese HCW [44], but lower than the overall rates for HCW found in a recent meta-analysis [10]. Focusing on the resources, comparisons between HCW revealed that nurses and physicians reported similar levels of SOC and social support. This is in line with previous findings [24, 45]. While pastoral workers reported high levels on all resources, especially on religiosity, MTA indicated the lowest resource levels of all four professions. This suggests that the focus of research should extend on MTA during the COVID-19 pandemic. First explanations like a lower socioeconomic position and high pressure have been provided why MTA in Germany might be the most vulnerable group during the COVID-19 pandemic [9]. However, more research among groups of HCW is necessary to obtain comparable data and to investigate if those differences in resources already existed before the COVID-19 pandemic.

Our main analysis revealed that strong SOC and high social support were associated with less anxiety and depression symptoms. In addition, more religious HCW reported fewer symptoms of depression, but not anxiety. SOC was by far the strongest predictor while social support and religiosity in particular showed only small effects. To the best of our knowledge, these three resources have not been investigated simultaneously in HCW before. Previous studies suggested that SOC may play a more influential role than social support for mental well-being. In a study among 372 nurses, SOC assessed with a three-item measure was a stronger predictor for general mental health than the social support [46]. Similar results emerged when scales of SOC (SOC-9) and resilience (RS-11) as another potential protective concept were used to predict mental health outcomes among ICU staff [24] and paramedics [23]. In primary care patients, social support even failed to show an effect on quality of life when SOC was accounted for [47]. Thus, as a rather inherent resource of HCW, perceiving life as coherent during the COVID-19 pandemic was linked to better mental health outcomes. This association was slightly more pronounced for anxiety than for depressive symptoms. However, considerations about the validity of the SOC have been raised [48] arguing that measurements of SOC and symptoms of depression and anxiety in particular are actually opposite sides of the same construct [49]. This conceptual overlap between SOC and mental health might explain the strong effects of SOC and the small effects of social support on mental symptoms in the multiple regression models, given that SOC and social support were also moderately correlated. However, it might also be possible that social support is not the most needed protective factor in the COVID-19 pandemic [50]. Although HCW reported normal levels of perceived social support, they may not have been able to use social contacts as effectively as before due to social restrictions during the pandemic or the fear of infecting close friends when meeting in person. Nevertheless, medium bivariate correlations with mental symptoms in this study and further associations in the body of literature [19, 51] demonstrate that social support is far from irrelevant. In the present sample, it tended to be more predictive of symptoms of depression than anxiety. Even though protective effects of social support might be smaller in times of the pandemic, they could still counteract feelings of loneliness and thus alleviate depression symptoms [52, 53]. Interestingly, social support was not significantly associated with better mental health outcomes among nurses and pastoral workers. Religiosity had only weak links to depression and none to anxiety in HCW. This tendency was consistent with findings from Kim et al. [31] showing that spirituality was associated with depression in multiple regression but not with anxiety. Nevertheless, the result should be interpreted with caution given the minimal effect sizes and the single item measurement of religiosity. Separate analyses of professional subgroups and work setting generally supported the previous findings. The lack of a significant effect of religiosity among pastoral workers in particular may be attributed to the comparatively small group size as well as limited variance in the item and thus a ceiling effect.

The resources explained substantially less variance in the increase of subjective burden than in mental health outcomes. Nevertheless, the results supported the importance of SOC, as HCW who perceive life as understandable and meaningful subjectively experienced lower increases in burden due to the pandemic. Contrary to our hypothesis, social support and religiosity seemed to be associated with a greater increase in burden in the total sample, even though social support was associated with less increase in burden in the bivariate correlations. In the sub analyses, social support failed to exhibit significant effects and religiosity had weak associations with mental health only in physicians and inpatient HCW. This suggests that the effects in the total sample, except for SOC, likely reached statistical significance in the regression as a result of the large sample rather than their relevance. An alternative, speculative idea might be that social support might have a double edge, as it also might mean to have more people to care for in the pandemic, or that social support is more valuated subjectively when the burden is higher.

### Limitations

The present study has several limitations. Firstly, for economic reasons we assessed data with short versions of the original instruments and single item measures. The PHQ-2 and GAD-2 have been well studied and widely used [33]. SOC-3 [34] and ESSI-D [36] were validated in previous studies but have not been used frequently in the literature which limits the comparability of the results. The SOC-3 in particular measures only two of the three original facets of SOC. Therefore, findings should be validated with longer SOC versions to include all three original facets. Additionally, using a revised scale focusing on relatedness and ambiguity [54] might overcome conceptual issues. Religiosity was measured with one item that also referred to spirituality. A single-item measurement is probably insufficient to capture multiple aspects of religiosity and its dependence on cultural influences. Therefore, future research should include more comprehensive measures of religiosity. Yet, in large samples, single items can help to provide initial insights into the research topic and set directions for subsequent research.

Secondly, due to the mode of data collection a potential selection bias of the sample has to be considered. However, participants were recruited through extensive employee portals which addressed all HCW in the respective hospital and for the university hospitals we were able to report estimates of the representativeness. Thus, we regard the study sample as partially representative of HCW from three professions (physicians, nurses, MTA, but probably not for pastoral workers) in Germany, at least for university hospitals.

Thirdly, causal interpretations cannot be drawn from the cross-sectional study design. Even though the data suggest that the resources attenuate mental health problems, other interpretations could be possible. For example, having the conceptual problems in mind, a low SOC might only mirror high levels of anxiety. Furthermore, suffering from feelings of depression could subsequently lead to less perceived social support. Eventually, only longitudinal studies will help to identify causal mechanisms of health-promoting resources. Data collection for longitudinal analyses is in progress and will be analyzed in further publications.

### Implications

Given that social support and SOC in particular showed strong associations with mental health during the COVID-19 pandemic, interventions that strengthen these resources and well-being should be tested and evaluated in HCW. Several resource- and resilience-related approaches have been identified in previous research that could potentially work beneficially. Among nurses, a mindfulness-based intervention did successfully enhance SOC [55]. Furthermore, physical activity has been associated with a stronger SOC [56]. These activities could also be carried out at home, in light of the restrictions imposed by the COVID-19 pandemic. Since the SOC is partly based on comprehensibility and meaningfulness, a rather COVID-19 specific approach might be to provide HCW with high levels of transparency concerning the measures taken in their work environment. However, further research is needed to investigate conceptual issues regarding the SOC-3 and mental health. As social interaction in person is reduced as a result of the pandemic, new approaches could help to maintain social support and use contacts as an effective resource. These can include telephone calls, video conferences or online platforms [57]. The findings also suggest that interventions and future research should particularly target MTA, which had the lowest levels of resources in the current study and were found to be a vulnerable group among HCW in Germany [9]. Finally, there is a need to explore why the importance of SOC, social support, and religiosity differs across professional groups, which in turn could help tailor intervention approaches for HCW.

## Conclusion

The current study identified the potential health-promoting effects of SOC and social support for HCW in the front line but also in the background during the COVID-19 pandemic in Germany. This is of particular interest given that these resources contribute in distinct ways to individual mental well-being and suggest different interventions. Future research should explore causal mechanisms of SOC and social support using longitudinal studies, while focusing on different medical professional groups.

## Supporting information

S1 File (DOCX)
