# Supplementary material for: Sense of coherence, social support and religiosity as resources for medical personnel during the COVID-19 pandemic: A web-based survey among 4324 health care workers within the German Network University Medicine
Source: PLoS One. 2021 Jul 26;16(7):e0255211. doi: 10.1371/journal.pone.0255211 (PMC8312980; doi:10.1371/journal.pone.0255211)
Supplement: S1 File — (DOCX) [file pone.0255211.s001.docx]

**S1 File - Table of Contents**

[Table 1. Linear regression analyses for general mental health symptoms (PHQ-4) for the total sample of healthcare workers. 2](#_Toc71023724)

[Table 2. Pairwise comparisons between medical professions. 3](#_Toc71023725)

[Table 3. Linear regression analyses for severity of depressive (PHQ-2) and generalized anxiety symptoms (GAD-2) for physicians. 4](#_Toc71023726)

[Table 4. Linear regression analyses for severity of depressive (PHQ-2) and generalized anxiety symptoms (GAD-2) for nurses. 5](#_Toc71023727)

[Table 5. Linear regression analyses for severity of depressive (PHQ-2) and generalized anxiety symptoms (GAD-2) for MTA. 6](#_Toc71023728)

[Table 6. Linear regression analyses for severity of depressive (PHQ-2) and generalized anxiety symptoms (GAD-2) for pastoral workers. 7](#_Toc71023729)

[Table 7. Linear regression analyses for severity of depressive (PHQ-2) and generalized anxiety symptoms (GAD-2) for HCW in the inpatient sector. 8](#_Toc71023730)

[Table 8. Linear regression analyses for severity of depressive (PHQ-2) and generalized anxiety symptoms (GAD-2) for HCW in the outpatient sector. 9](#_Toc71023731)

[Table 9. Linear regression analysis for increase in burden for physicians and nurses. 10](#_Toc71023732)

[Table 10. Linear regression analysis for increase in burden for MTA and pastoral workers. 11](#_Toc71023733)

[Table 11. Linear regression analysis for increase in burden for the two work settings of healthcare workers. 12](#_Toc71023734)

# Table 1. Linear regression analyses for general mental health symptoms (PHQ-4) for the total sample of healthcare workers.

| Independent Variables |  |  | PHQ-4 (*F*(12, 4311) = 277.023, *p* < .001, adjusted *R*² = .434) | | | | |
| --- | --- | --- | --- | --- | --- | --- | --- |
|  | *R²* | ∆ *R²* | B (95% CI) | SE | β | *t* | *p* |
| **Step 1**: Control Variables | .013 | .013 |  |  |  |  |  |
| Gender^a^ |  |  | .211 [.068, .353] | .072 | .034 | 2.905 | **.004** |
| Age^b^ |  |  |  |  |  |  |  |
| 31-40 |  |  | .002 [-.219, .223] | .113 | .000 | .021 | .984 |
| 41-50 |  |  | .048 [-.191, .286] | .122 | .007 | .392 | .695 |
| 51-60 |  |  | .273 [.039, .507] | .119 | .045 | 2.284 | **.022** |
| >60 |  |  | -.040 [-.348, .269] | .157 | -.004 | -.252 | .801 |
| Professional experience^c^ |  |  |  |  |  |  |  |
| 3-6 years |  |  | .028 [-.267, .323] | .150 | .003 | .185 | .853 |
| >6 years |  |  | .337 [.062, .612] | .140 | .056 | 2.405 | **.016** |
| unknown |  |  | .285 [-.030, .599] | .160 | .033 | 1.773 | .076 |
| Contact with   SARS-CoV-2^d^ |  |  | .056 [-.072, .184] | .065 | .010 | .863 | .388 |
| **Step 2**: Resources | .435 | .422 |  |  |  |  |  |
| Sense of Coherence |  |  | -.456 [-.474, -.437] | .009 | -.628 | -48.944 | **<.001** |
| Social Support |  |  | -.049 [-.066, -.032] | .009 | -.072 | -5.671 | **<.001** |
| Religiosity |  |  | -.016 [-.075, .043] | .030 | -.006 | -.518 | .605 |

*Notes.* SE = Standard Error. ^a^ Reference group = male. ^b^ Reference group = Age 18 -30. ^c^ Reference group = Professional experience <3 years. ^d^ Having contact with either COVID-19 infected patients or contaminated material; Reference group = no.

# Table 2. Pairwise comparisons between medical professions.

| Dependent variable | Profession (I) | Profession (J) | Difference  (I-J) | *t* | *df* | *p* | *d* |
| --- | --- | --- | --- | --- | --- | --- | --- |
| PHQ-2 | Physician | Nurse | -.19 | -3.38 | 2441 | .001 | -.13 |
|  |  | MTA | -.40 | -7.42 | 2982 | **<.001** | -.27 |
|  |  | PW | .30 | 3.24 | 210 | .001 | .22 |
|  | Nurse | MTA | -.21 | -3.51 | 2548 | **<.001** | -.14 |
|  |  | PW | .49 | 5.13 | 237 | **<.001** | .34 |
|  | MTA | PW | .70 | 7.42 | 221 | **<.001** | .47 |
| GAD-2 | Physician | Nurse | .05 | .85 | 2491 | .394 | .03 |
|  |  | MTA | -.19 | -3.34 | 2992 | .001 | -.12 |
|  |  | PW | .39 | 4.07 | 215 | **<.001** | .27 |
|  | Nurse | MTA | -.24 | -3.95 | 2563 | **<.001** | -.15 |
|  |  | PW | .34 | 3.46 | 237 | .001 | .23 |
|  | MTA | PW | .58 | 5.98 | 223 | **<.001** | .37 |
| SOC-3 | Physician | Nurse | .04 | .30 | 2495 | .768 | .01 |
|  |  | MTA | 1.04 | 7.53 | 2987 | **<.001** | .28 |
|  |  | PW | -1.18 | -4.62 | 201 | **<.001** | -.33 |
|  | Nurse | MTA | 1.00 | 6.75 | 2581 | **<.001** | .26 |
|  |  | PW | -1.23 | -4.69 | 218 | **<.001** | -.34 |
|  | MTA | PW | -2.22 | -8.59 | 209 | **<.001** | -.58 |
| ESSI-D | Physician | Nurse | -.18 | -1.19 | 2464 | .236 | -.05 |
|  |  | MTA | .56 | 3.83 | 2974 | **<.001** | .14 |
|  |  | PW | -.23 | -.71 | 183 | .477 | -.06 |
|  | Nurse | MTA | .74 | 4.67 | 2583 | **<.001** | .18 |
|  |  | PW | -.05 | -.15 | 196 | .878 | -.01 |
|  | MTA | PW | -.79 | -2.42 | 190 | .016 | -.19 |
| Religiosity | Physician | Nurse | .21 | 5.11 | 2511 | **<.001** | .20 |
|  |  | MTA | .24 | 6.25 | 2992 | **<.001** | .23 |
|  |  | PW | -1.47 | -27.20 | 267 | **<.001** | -1.45 |
|  | Nurse | MTA | .03 | .64 | 2465 | .524 | .03 |
|  |  | PW | -1.68 | -30.03 | 302 | **<.001** | -1.67 |
|  | MTA | PW | -1.70 | -31.88 | 256 | **<.001** | -1.75 |

*Notes*. Welch’s *t*-tests with Bonferroni adjusted α-level (α = 0.05/6 = 0.008) were conducted for pairwise comparisons. Therefore *p*-values < .008 indicate significant mean difference.

# Table 3. Linear regression analyses for severity of depressive (PHQ-2) and generalized anxiety symptoms (GAD-2) for physicians.

| Independent Variables | Model 1: PHQ-2 (*F*(12, 1479) = 72.755, *p* < .001, adjusted *R*² = .366) | | | | | | | Model 2: GAD-2 (*F*(12, 1479) = 80.423, *p* < .001, adjusted *R*² = .390) | | | | | | |
| --- | --- | --- | --- | --- | --- | --- | --- | --- | --- | --- | --- | --- | --- | --- |
|  | *R²* | ∆ *R²* | B (95% CI) | SE | Beta | *t* | *p* | *R²* | ∆ *R²* | B (95% CI) | SE | Beta | *t* | *p* |
|  |  |  |  |  |  |  |  |  |  |  |  |  |  |  |
| Step 1: Control Variables | .013 | .013 |  |  |  |  |  | .029 | .029 |  |  |  |  |  |
| Gender^a^ |  |  | .021 [-.098, .140] | .061 | .007 | .345 | .730 |  |  | .196 [.071, .322] | .064 | .064 | 3.072 | .002 |
| Age^b^ |  |  |  |  |  |  |  |  |  |  |  |  |  |  |
| 31-40 |  |  | .200 [-.104, .504] | .155 | .063 | 1.290 | .197 |  |  | .181 [-.139, .501] | .163 | .053 | 1.109 | .268 |
| 41-50 |  |  | .166 [-.178, .510] | .175 | .051 | .946 | .345 |  |  | .272 [-.090, .634] | .185 | .077 | 1.474 | .141 |
| 51-60 |  |  | .254 [-.089, .598] | .175 | .082 | 1.453 | .146 |  |  | .424 [.062, .786] | .184 | .128 | 2.300 | **.022** |
| >60 |  |  | .114 [-.255, .483] | .188 | .026 | .605 | .545 |  |  | .100 [-.289, .489] | .198 | .021 | .506 | .613 |
| Professional experience^c^ |  |  |  |  |  |  |  |  |  |  |  |  |  |  |
| 3-6 years |  |  | -.057 [-.363, .249] | .156 | -.012 | -.368 | .713 |  |  | -.337 [-.659, -.015] | .164 | -.066 | -2.051 | **.040** |
| >6 years |  |  | -.118 [-.445, .210] | .167 | -.035 | -.704 | .482 |  |  | -.089 [-.434, .257] | .176 | -.024 | -.504 | .615 |
| unknown |  |  | -.242 [-.684, .200] | .225 | -.032 | -1.075 | .283 |  |  | -.307 [-.773, .158] | .237 | -.038 | -1.295 | .196 |
| Contact with SARS-CoV-2^d^ |  |  | .011 [-.105, .127] | .059 | .004 | .192 | .848 |  |  | .050 [-.073, .172] | .062 | .016 | .795 | .427 |
| Step 2: Resources | .371 | .358 |  |  |  |  |  | .395 | .365 |  |  |  |  |  |
| Sense of Coherence |  |  | -.214 [-.231, -.196] | .009 | -.556 | -23.990 | **<.001** |  |  | -.239 [-.257, -.220] | .009 | -.578 | -25.428 | **<.001** |
| Social Support |  |  | -.035 [-.052, -.018] | .009 | -.095 | -4.118 | **<.001** |  |  | -.030 [-.047, -.012] | .009 | -.074 | -3.275 | **.001** |
| Religiosity |  |  | -.039 [-.094, -.016] | .028 | -.029 | -1.390 | .165 |  |  | .016 [-.042, .074] | .030 | .011 | .550 | .583 |

*Notes.* ∆ *R²* = Change in *R².* SE = Standard Error. ^a^ Reference group = male. ^b^ Reference group = Age 18 -30. ^c^ Reference group = Professional experience <3 years. ^d^ Having contact with either COVID-19 infected patients or contaminated material; Reference group = no.

# Table 4. Linear regression analyses for severity of depressive (PHQ-2) and generalized anxiety symptoms (GAD-2) for nurses.

| Independent Variables | Model 1: PHQ-2 (*F*(12, 1158) = 45.708, *p* < .001, adjusted *R*² = .314) | | | | | | | Model 2: GAD-2 (*F*(12, 1158) = 54.360, *p* < .001, adjusted *R*² = .354) | | | | | | |
| --- | --- | --- | --- | --- | --- | --- | --- | --- | --- | --- | --- | --- | --- | --- |
|  | *R²* | ∆ *R²* | B (95% CI) | SE | Beta | *t* | *p* | *R²* | ∆ *R²* | B (95% CI) | SE | Beta | *t* | *p* |
|  |  |  |  |  |  |  |  |  |  |  |  |  |  |  |
| *Step 1: Control Variables* | .014 | .014 |  |  |  |  |  | .011 | .011 |  |  |  |  |  |
| Gender^a^ |  |  | -.034 [-.202, .134] | .086 | -.010 | -.397 | .692 |  |  | .054 [-.202, .134] | .086 | .015 | .634 | .526 |
| Age^b^ |  |  |  |  |  |  |  |  |  |  |  |  |  |  |
| 31-40 |  |  | -.177 [-.417, .064] | .123 | -.052 | -1.441 | .150 |  |  | .212 [-.417, .064] | .123 | .060 | 1.724 | .085 |
| 41-50 |  |  | -.258 [-.514, -.002] | .131 | -.072 | -1.976 | **.048** |  |  | .281 [-.514, -.002] | .131 | .075 | 2.145 | **.032** |
| 51-60 |  |  | -.149 [-.409, .110] | .132 | -.042 | -1.128 | .260 |  |  | .292 [-.409, .110] | .133 | .079 | 2.200 | **.028** |
| >60 |  |  | -.327 [-.736, .081] | .208 | -.043 | -1.573 | .116 |  |  | .379 [-.736, .081] | .208 | .049 | 1.816 | .070 |
| Professional experience^c^ |  |  |  |  |  |  |  |  |  |  |  |  |  |  |
| 3-6 years |  |  | .024 [-.313, .361] | .172 | .006 | .142 | .887 |  |  | -.153 [-.313, .361] | .172 | -.034 | -.887 | .375 |
| >6 years |  |  | .235 [-.100, .570] | .171 | .066 | 1.378 | .169 |  |  | .067 [-.100, .570] | .171 | .018 | .393 | .695 |
| unknown |  |  | .195 [-.379, .770] | .293 | .020 | .667 | .505 |  |  | .207 [-.379, .770] | .293 | .020 | .705 | .481 |
| Contact with SARS-CoV-2^d^ |  |  | -.059 [-.204, .086] | .074 | -.020 | -.795 | .427 |  |  | .155 [-.204, .086] | .074 | .050 | 2.095 | **.036** |
| *Step 2: Resources* | .321 | .308 |  |  |  |  |  | .360 | .349 |  |  |  |  |  |
| Sense of Coherence |  |  | -.220 [-.242, -.199] | .011 | -.548 | -20.154 | **<.001** |  |  | -.251 [-.272, -.229] | .011 | -.605 | -22.924 | **<.001** |
| Social Support |  |  | -.014 [-.034, .006] | .010 | -.038 | -1.396 | .163 |  |  | .003 [-.017, .023] | .010 | .007 | .268 | .789 |
| Religiosity |  |  | -.035 [-.104, .034] | .035 | -.025 | -1.007 | .314 |  |  | .065 [-.004, .134] | .035 | .045 | 1.856 | .064 |

*Notes.* ∆ *R²* = Change in *R².* SE = Standard Error. ^a^ Reference group = male. ^b^ Reference group = Age 18 -30. ^c^ Reference group = Professional experience <3 years. ^d^ Having contact with either COVID-19 infected patients or contaminated material; Reference group = no.

# Table 5. Linear regression analyses for severity of depressive (PHQ-2) and generalized anxiety symptoms (GAD-2) for MTA.

| Independent Variables | Model 1: PHQ-2 (*F*(12, 1496) = 60.473, *p* < .001, adjusted *R*² = .321) | | | | | | | Model 2: GAD-2 (*F*(12, 1496) = 88.120, *p* < .001, adjusted *R*² = .409) | | | | | | |
| --- | --- | --- | --- | --- | --- | --- | --- | --- | --- | --- | --- | --- | --- | --- |
|  | *R²* | ∆ *R²* | B (95% CI) | SE | Beta | *t* | *p* | *R²* | ∆ *R²* | B (95% CI) | SE | Beta | *t* | *p* |
|  |  |  |  |  |  |  |  |  |  |  |  |  |  |  |
| Step 1: Control variables | .012 | .012 |  |  |  |  |  | .010 | .010 |  |  |  |  |  |
| Gender^a^ |  |  | -.013 [-.209, .183] | .100 | -.003 | -.130 | .897 |  |  | .292 [.101, .483] | .097 | .061 | 3.004 | **.003** |
| Age^b^ |  |  |  |  |  |  |  |  |  |  |  |  |  |  |
| 31-40 |  |  | -.105 [-.330, .121] | .115 | -.029 | -.911 | .363 |  |  | -.037 [-.257, .186] | .112 | -.010 | -.329 | .742 |
| 41-50 |  |  | -.140 [-.369, .089] | .117 | -.039 | -1.200 | .230 |  |  | .180 [-.044, .403] | .114 | .047 | 1.579 | .115 |
| 51-60 |  |  | .004 [-.223, .232] | .116 | .001 | .036 | .971 |  |  | .412 [.190, .634] | .113 | .117 | 3.640 | **.000** |
| >60 |  |  | -.002 [-.352, .347] | .178 | .000 | -.014 | .989 |  |  | .380 [.038, .721] | .174 | .051 | 2.181 | **.029** |
| Professional experience^c^ |  |  |  |  |  |  |  |  |  |  |  |  |  |  |
| 3-6 years |  |  | .340 [.004, .677] | .172 | .061 | 1.984 | **.047** |  |  | .051 [-.278, .379] | .167 | .009 | .302 | .763 |
| >6 years |  |  | .401 [.101, .701] | .153 | .129 | 2.619 | **.009** |  |  | .240 [-.052, .533] | .149 | .074 | 1.610 | .108 |
| unknown |  |  | .465 [.160, .769] | .155 | .132 | 2.993 | **.003** |  |  | .236 [-.062, .533] | .152 | .064 | 1.555 | .120 |
| Contact with SARS-CoV-2^d^ |  |  | -.115 [-.263, .034] | .076 | -.032 | -1.516 | .130 |  |  | .094 [-.051, .239] | .074 | .025 | 1.275 | .203 |
| Step 2: Resources | .327 | .315 |  |  |  |  |  | .414 | .404 |  |  |  |  |  |
| Sense of Coherence |  |  | -.206 [-.224, -.188] | .009 | -.528 | -22.356 | **<.001** |  |  | -.252 [-.270, -.234] | .009 | -.619 | -28.080 | **<.001** |
| Social Support |  |  | -.036 [-.052, -.019] | .008 | -.098 | -4.222 | **<.001** |  |  | -.029 [-.045, -.012] | .008 | -.075 | -3.476 | **.001** |
| Religiosity |  |  | -.038 [-.102, .027] | .033 | -.025 | -1.146 | .252 |  |  | .010 [-.053, .073] | .032 | .006 | .315 | .753 |

*Notes.* ∆ *R²* = Change in *R².* SE = Standard Error. ^a^ Reference group = male. ^b^ Reference group = Age 18 -30. ^c^ Reference group = Professional experience <3 years. ^d^ Having contact with either COVID-19 infected patients or contaminated material; Reference group = no.

# Table 6. Linear regression analyses for severity of depressive (PHQ-2) and generalized anxiety symptoms (GAD-2) for pastoral workers.

| Independent Variables | Model 1: PHQ-2 (*F*(11, 140) = 4.418, *p* < .001, adjusted *R*² = .200) | | | | | | | Model 2: GAD-2 (*F*(11, 140) = 7.385, *p* < .001, adjusted *R*² = .317) | | | | | | |
| --- | --- | --- | --- | --- | --- | --- | --- | --- | --- | --- | --- | --- | --- | --- |
|  | *R²* | ∆ *R²* | B (95% CI) | SE | Beta | *t* | *p* | *R²* | ∆ *R²* | B (95% CI) | SE | Beta | *t* | *p* |
|  |  |  |  |  |  |  |  |  |  |  |  |  |  |  |
| Step 1: Control variables | .057 | .005 |  |  |  |  |  | .162 | .162 |  |  |  |  |  |
| Gender^a^ |  |  | -.046 [-.368, .275] | .163 | -.022 | -.284 | .777 |  |  | .481 [.174, .788] | .155 | .220 | 3.099 | **.002** |
| Age^b^ |  |  |  |  |  |  |  |  |  |  |  |  |  |  |
| 31-40 |  |  | 1.133 [-.090, 2.36] | .618 | .151 | 1.832 | .069 |  |  | 1.623 [.456, 2.789] | .590 | .209 | 2.751 | **.007** |
| 41-50 |  |  | .399 [-.170, .968] | .288 | .126 | 1.386 | .168 |  |  | .039 [-.504, .582] | .275 | .012 | .142 | .887 |
| 51-60 |  |  | .038 [-.347, .422] | .194 | .018 | .194 | .846 |  |  | .190 [-.177, .557] | .186 | .085 | 1.023 | .308 |
| Professional experience^c^ |  |  |  |  |  |  |  |  |  |  |  |  |  |  |
| 3-6 years |  |  | .106 [-.505, .717] | .309 | .034 | .343 | .732 |  |  | .265 [-.318, .847] | .295 | .081 | .899 | .370 |
| >6 years |  |  | .258 [-.223, .739] | .243 | .123 | 1.059 | .291 |  |  | .421 [-.038, .880] | .232 | .194 | 1.813 | .072 |
| unknown |  |  | .024 [-.548, .596] | .289 | .009 | .083 | .934 |  |  | -.108 [-.653, .438] | .276 | -.039 | -.390 | .697 |
| Contact with SARS-CoV-2^d^ |  |  | -.218 [-.529, .094] | .158 | -.104 | -1.379 | .170 |  |  | .072 [-.226, .369] | .151 | .033 | .476 | .635 |
| Step 2: Resources | .258 | .199 |  |  |  |  |  | .367 | .205 |  |  |  |  |  |
| Sense of Coherence |  |  | -.138 [-.196, -.079] | .029 | -.385 | -4.683 | **<.001** |  |  | -.153 [-.209, -.098] | .028 | -.415 | -5.469 | **<.001** |
| Social Support |  |  | -.040 [-.086, .005] | .023 | -.146 | -1.767 | .079 |  |  | -.031 [-.074, .012] | .022 | -.108 | -1.424 | .157 |
| Religiosity |  |  | .090 [-.189, .369] | .141 | .050 | .641 | .523 |  |  | .111 [-.156, .377] | .135 | .059 | .821 | .413 |

*Notes.* ∆ *R²* = Change in *R².* SE = Standard Error. ^a^ Reference group = male. ^b^ Reference group = Age 18 -30. ^c^ Reference group = Professional experience <3 years. ^d^ Having contact with either COVID-19 infected patients or contaminated material; Reference group = no.

#

# Table 7. Linear regression analyses for severity of depressive (PHQ-2) and generalized anxiety symptoms (GAD-2) for HCW in the inpatient sector.

| Independent Variables | Model 1: PHQ-2 (*F*(12, 3483) = 146.015, *p* < .001, adjusted *R*² = .332) | | | | | | | Model 2: GAD-2 (*F*(12, 3483) = 172.779, *p* < .001, adjusted *R*² = .371) | | | | | | |
| --- | --- | --- | --- | --- | --- | --- | --- | --- | --- | --- | --- | --- | --- | --- |
|  | *R²* | ∆ *R²* | B (95% CI) | SE | Beta | *t* | *p* | *R²* | ∆ *R²* | B (95% CI) | SE | Beta | *t* | *p* |
|  |  |  |  |  |  |  |  |  |  |  |  |  |  |  |
| Step 1: Control variables | .016 | .016 |  |  |  |  |  | .014 | .014 |  |  |  |  |  |
| Gender^a^ |  |  | .021 [-.071, .114] | .047 | .006 | .454 | .650 |  |  | .151 [.058, .243] | .047 | .044 | 3.197 | **.001** |
| Age^b^ |  |  |  |  |  |  |  |  |  |  |  |  |  |  |
| 31-40 |  |  | -.118 [-.256, .019] | .070 | -.035 | -1.685 | .092 |  |  | .126 [-.012, .264] | .070 | .036 | 1.785 | .074 |
| 41-50 |  |  | -.175 [-.325, -.024] | .077 | -.050 | -2.277 | **.023** |  |  | .245 [.094, .396] | .077 | .067 | 3.192 | **.001** |
| 51-60 |  |  | -.083 [-.231, .065] | .076 | -.025 | -1.096 | .273 |  |  | .326 [.178, .475] | .076 | .095 | 4.307 | **<.001** |
| >60 |  |  | -.286 [-.499, -.073] | .109 | -.045 | -2.628 | **.009** |  |  | .153 [-.060, .367] | .109 | .023 | 1.409 | .159 |
| Professional experience^c^ |  |  |  |  |  |  |  |  |  |  |  |  |  |  |
| 3-6 years |  |  | .162 [-.018, .342] | .092 | .035 | 1.764 | .078 |  |  | -.151 [-.332, .029] | .092 | -.032 | -1.649 | .099 |
| >6 years |  |  | .268 [.099, .437] | .086 | .085 | 3.111 | **.002** |  |  | .049 [-.120, .218] | .086 | .015 | .563 | .573 |
| unknown |  |  | .315 [.118, .513] | .101 | .068 | 3.136 | **.002** |  |  | -.022 [-.219, .176] | .101 | -.005 | -.216 | .829 |
| Contact with SARS-CoV-2^d^ |  |  | -.007 [-.091, .076] | .043 | -.002 | -.167 | .867 |  |  | .095 [.012, .179] | .043 | .030 | 2.233 | **.026** |
| Step 2: Resources | .335 | .319 |  |  |  |  |  | .373 | .359 |  |  |  |  |  |
| Sense of Coherence |  |  | -.210 [-.222, -.198] | .006 | -.531 | -34.187 | **<.001** |  |  | -.241 [-.253, -.229] | .006 | -.591 | -39.205 | **<.001** |
| Social Support |  |  | -.033 [-.045, -.022] | .006 | -.089 | -5.794 | **<.001** |  |  | -.019 [-.030, -.008] | .006 | -.049 | -3.268 | **.001** |
| Religiosity |  |  | -.043 [-.082, -.004] | .020 | -.031 | -2.184 | **.029** |  |  | .033 [-.006, .071] | .020 | .023 | 1.659 | .097 |

*Notes.* ∆ *R²* = Change in *R².* SE = Standard Error. ^a^ Reference group = male. ^b^ Reference group = Age 18 -30. ^c^ Reference group = Professional experience <3 years. ^d^ Having contact with either COVID-19 infected patients or contaminated material; Reference group = no.

# Table 8. Linear regression analyses for severity of depressive (PHQ-2) and generalized anxiety symptoms (GAD-2) for HCW in the outpatient sector.

| Independent Variables | Model 1: PHQ-2 (*F*(12, 815) = 42.230, *p* < .001, adjusted *R*² = .374) | | | | | | | Model 2: GAD-2 (*F*(12, 815) = 54.257, *p* < .001, adjusted *R*² = .436) | | | | | | |
| --- | --- | --- | --- | --- | --- | --- | --- | --- | --- | --- | --- | --- | --- | --- |
|  | *R²* | ∆ *R²* | B (95% CI) | SE | Beta | *t* | *p* | *R²* | ∆ *R²* | B (95% CI) | SE | Beta | *t* | *p* |
|  |  |  |  |  |  |  |  |  |  |  |  |  |  |  |
| Step 1: Control variables | .024 | .024 |  |  |  |  |  | .035 | .035 |  |  |  |  |  |
| Gender^a^ |  |  | .082 [-.101, .266] | .093 | .025 | .883 | .378 |  |  | .310 [.119, .501] | .097 | .087 | 3.189 | **.001** |
| Age^b^ |  |  |  |  |  |  |  |  |  |  |  |  |  |  |
| 31-40 |  |  | -.060 [-.419, .299] | .183 | -.015 | -.327 | .744 |  |  | .033 [-.341, .406] | .190 | .008 | .173 | .863 |
| 41-50 |  |  | -.136 [-.508, .235] | .189 | -.039 | -.721 | .471 |  |  | .132 [-.255, .518] | .197 | .034 | .668 | .504 |
| 51-60 |  |  | -.036 [-.403, .331] | .187 | -.012 | -.190 | .848 |  |  | .431 [.050, .813] | .194 | .128 | 2.219 | **.027** |
| >60 |  |  | -.014 [-.422, .395] | .208 | -.003 | -.066 | .947 |  |  | .213 [-.212, .638] | .216 | .048 | .984 | .325 |
| Professional experience^c^ |  |  |  |  |  |  |  |  |  |  |  |  |  |  |
| 3-6 years |  |  | .170 [-.413, .752] | .297 | .026 | .572 | .568 |  |  | .130 [-.476, .736] | .309 | .018 | .421 | .674 |
| >6 years |  |  | .172 [-.350, .695] | .266 | .046 | .647 | .518 |  |  | .449 [-.095, .992] | .277 | .110 | 1.621 | .105 |
| unknown |  |  | .233 [-.312, .777] | .277 | .050 | .839 | .402 |  |  | .278 [-.289, .844] | .288 | .054 | .963 | .336 |
| Contact with SARS-CoV-2^d^ |  |  | -.143 [-.308, .021] | .084 | -.048 | -1.709 | .088 |  |  | .104 [-.067, .275] | .087 | .032 | 1.189 | .235 |
| Step 2: Resources | .383 | .359 |  |  |  |  |  | .444 | .409 |  |  |  |  |  |
| Sense of Coherence |  |  | -.217 [-.240, -.195] | .012 | -.586 | -18.778 | **<.001** |  |  | -.258 [-.282, -.234] | .012 | -.635 | -21.429 | **<.001** |
| Social Support |  |  | -.021 [-.042, .000] | .011 | -.060 | -1.974 | **.049** |  |  | -.021 [-.042, .001] | .011 | -.054 | -1.864 | .063 |
| Religiosity |  |  | -.033 [-.107, .042] | .038 | -.025 | -.867 | .386 |  |  | .007 [-.071, .084] | .039 | .004 | .166 | .868 |

*Notes.* ∆ *R²* = Change in *R².* SE = Standard Error. ^a^ Reference group = male. ^b^ Reference group = Age 18 -30. ^c^ Reference group = Professional experience <3 years. ^d^ Having contact with either COVID-19 infected patients or contaminated material; Reference group = no.

# Table 9. Linear regression analysis for increase in burden for physicians and nurses.

| Independent Variables | Physicians: Increase in Burden (*F*(12, 1479) = 10.117, *p* < .001, adjusted *R*² = .068) | | | | | | |
| --- | --- | --- | --- | --- | --- | --- | --- |
|  | *R²* | ∆ *R²* | B (95% CI) | SE | Beta | *t* | *p* |
| Step 1: Control variables | .040 | .040 |  |  |  |  |  |
| Gender^a^ |  |  | .092 [-.042, .225] | .068 | .035 | 1.347 | .178 |
| Age^b^ |  |  |  |  |  |  |  |
| 31-40 |  |  | -.095 [-.436, .246] | .174 | -.032 | -.546 | .585 |
| 41-50 |  |  | .188 [-.197, .574] | .197 | .062 | .958 | .338 |
| 51-60 |  |  | .335 [-.050, .720] | .196 | .117 | 1.709 | .088 |
| >60 |  |  | .406 [-.008, .820] | .211 | .101 | 1.926 | .054 |
| Professional experience^c^ |  |  |  |  |  |  |  |
| 3-6 years |  |  | .062 [-.281, .405] | .175 | .014 | .354 | .723 |
| >6 years |  |  | .352 [-.015, .720] | .187 | .113 | 1.881 | .060 |
| unknown |  |  | .297 [-.198, .793] | .253 | .042 | 1.117 | .239 |
| Contact with SARS-CoV-2^d^ |  |  | .094 [-.036, .224] | .066 | .036 | 1.418 | .156 |
| Step 2: Resources | .076 | .036 |  |  |  |  |  |
| Sense of Coherence |  |  | -.067 [-.087, -.047] | .010 | -.189 | -6.714 | **<.001** |
| Social Support |  |  | .000 [-.019, .019] | .010 | .001 | .020 | .984 |
| Religiosity |  |  | .071 [.009, .132] | .031 | .057 | 2.246 | **.025** |
|  | Nurses: Increase in Burden (*F*(12, 1158) = 3.102, *p* < .001, adjusted *R*² = .021) | | | | | | |
|  | *R²* | ∆ *R²* | B (95% CI) | SE | Beta | *t* | *p* |
| Step 1: Control variables | .016 | .016 |  |  |  |  |  |
| Gender^a^ |  |  | .172 [.005, .339] | .085 | .059 | 2.022 | **.043** |
| Age^b^ |  |  |  |  |  |  |  |
| 31-40 |  |  | -.153 [-.392, .086] | .122 | -.054 | -1.256 | .209 |
| 41-50 |  |  | .084 [-.171, .339] | .130 | .028 | .646 | .519 |
| 51-60 |  |  | .019 [-.239, .277] | .132 | .006 | .145 | .885 |
| >60 |  |  | .097 [-.309, .502] | .207 | .015 | .468 | .640 |
| Professional experience^c^ |  |  |  |  |  |  |  |
| 3-6 years |  |  | .025 [-.309, .360] | .171 | .007 | .148 | .883 |
| >6 years |  |  | .288 [-.045, .620] | .170 | .098 | 1.695 | .090 |
| unknown |  |  | .058 [-.513 .629] | .291 | .007 | .200 | .842 |
| Contact with SARS-CoV-2^d^ |  |  | -.022 [-.166, .122] | .073 | -.009 | -.305 | .761 |
| Step 2: Resources | .031 | .015 |  |  |  |  |  |
| Sense of Coherence |  |  | -.041 [-.063, -.020] | .011 | -.124 | -3.807 | **<.001** |
| Social Support |  |  | .018 [-.002, .038] | .010 | .058 | 1.779 | .075 |
| Religiosity |  |  | .066 [-.002, .134] | .035 | .056 | 1.892 | .059 |

*Notes.* ∆ *R²* = Change in *R².* SE = Standard Error. ^a^ Reference group = male. ^b^ Reference group = Age 18 -30. ^c^ Reference group = Professional experience <3 years. ^d^ Reference group = no.

# Table 10. Linear regression analysis for increase in burden for MTA and pastoral workers.

| Independent Variables | MTA: Increase in Burden (*F*(12, 1496) = 4.084, *p* < .001, adjusted *R*² = .024) | | | | | | |
| --- | --- | --- | --- | --- | --- | --- | --- |
|  | *R²* | ∆ *R²* | B (95% CI) | SE | Beta | *t* | *p* |
| Step 1: Control variables | .008 | .008 |  |  |  |  |  |
| Gender^a^ |  |  | .111 [-.075, .298] | .095 | .030 | 1.172 | .241 |
| Age^b^ |  |  |  |  |  |  |  |
| 31-40 |  |  | .124 [-.091, .338] | .109 | .043 | 1.129 | .259 |
| 41-50 |  |  | .270 [.052, .488] | .111 | .094 | 2.431 | .015 |
| 51-60 |  |  | .144 [-.072, .361] | .110 | .054 | 1.306 | .192 |
| >60 |  |  | .419 [.086, .753] | .170 | .074 | 2.469 | .014 |
| Professional experience^c^ |  |  |  |  |  |  |  |
| 3-6 years |  |  | .260 [-.060, .581] | .163 | .059 | 1.592 | .112 |
| >6 years |  |  | .186 [-.099, .472] | .146 | .075 | 1.279 | .201 |
| unknown |  |  | .191 [-.099, .481] | .148 | .068 | 1.292 | .197 |
| Contact with SARS-CoV-2^d^ |  |  | .070 [-.072, .211] | .072 | .025 | .967 | .334 |
| Step 2: Resources | .032 | .023 |  |  |  |  |  |
| Sense of Coherence |  |  | -.052 [-.069, -.035] | .009 | -.168 | -5.938 | **<.001** |
| Social Support |  |  | .016 [.000, .031] | .008 | .054 | 1.940 | .053 |
| Religiosity |  |  | .032 [-.030, .093] | .031 | .026 | 1.012 | .312 |
|  | Pastoral workers: Increase in Burden (*F*(11, 140) = .401, *p* = .954, adjusted *R*² = -.046) | | | | | | |
|  | *R²* | ∆ *R²* | B (95% CI) | SE | Beta | *t* | *p* |
| Step 1: Control variables | .017 | .017 |  |  |  |  |  |
| Gender^a^ |  |  | .195 [-.179, .568] | .189 | .091 | 1.031 | **.304** |
| Age^b^ |  |  |  |  |  |  |  |
| 31-40 |  |  | -.072 [-1.49, 1.35] | .717 | -.009 | -.100 | **.920** |
| 41-50 |  |  | -.069 [-.730, .591] | .334 | -.022 | -.208 | .836 |
| 51-60 |  |  | -.049 [-.495, .397] | .226 | -.022 | -.216 | .829 |
| Professional experience^c^ |  |  |  |  |  |  |  |
| 3-6 years |  |  | .118 [-.590, .827] | .358 | .037 | .330 | .742 |
| >6 years |  |  | -.070 [-.628, .488] | .282 | -.033 | -.249 | .804 |
| unknown |  |  | -.003 [-.666, .661] | .336 | -.001 | -.008 | .994 |
| Contact with SARS-CoV-2^d^ |  |  | .215 [-.147, .576] | .183 | .101 | 1.173 | .243 |
| Step 2: Resources | .031 | .013 |  |  |  |  |  |
| Sense of Coherence |  |  | -.032 [-.100, .035] | .034 | -.089 | -.948 | .345 |
| Social Support |  |  | .034 [-.018, .087] | .027 | .122 | 1.292 | .199 |
| Religiosity |  |  | .007 [-.317, .330] | .164 | .004 | .040 | .968 |

*Notes.* ∆ *R²* = Change in *R².* SE = Standard Error. ^a^ Reference group = male. ^b^ Reference group = Age 18 -30. ^c^ Reference group = Professional experience <3 years. ^d^ Reference group = no.

# Table 11. Linear regression analysis for increase in burden for the two work settings of healthcare workers.

| Independent Variables | Inpatient: Increase in Burden (*F*(12, 3483) = 10.137, *p* < .001, adjusted *R*² = .030) | | | | | | |
| --- | --- | --- | --- | --- | --- | --- | --- |
|  | *R²* | ∆ *R²* | B (95% CI) | SE | Beta | *t* | *p* |
| Step 1: Control variables | .014 | .014 |  |  |  |  |  |
| Gender^a^ |  |  | .119 [.024, .214] | .049 | .041 | 2.453 | **.014** |
| Age^b^ |  |  |  |  |  |  |  |
| 31-40 |  |  | -.022 [-.164, .120] | .072 | -.008 | -.308 | .758 |
| 41-50 |  |  | .172 [.017, .327] | .079 | .057 | 2.177 | **.030** |
| 51-60 |  |  | .126 [-.027, .279] | .078 | .044 | 1.619 | .106 |
| >60 |  |  | .298 [.078, .518] | .112 | .055 | 2.659 | **.008** |
| Professional experience^c^ |  |  |  |  |  |  |  |
| 3-6 years |  |  | .095 [-.090, .281] | .095 | .024 | 1.009 | .313 |
| >6 years |  |  | .254 [.080, .428] | .089 | .094 | 2.864 | **.004** |
| unknown |  |  | .271 [.068, .474] | .104 | .068 | 2.614 | **.009** |
| Contact with SARS-CoV-2^d^ |  |  | .082 [-.005, .168] | .044 | .031 | 1.857 | .063 |
| Step 2 | .034 | .020 |  |  |  |  |  |
| Sense of Coherence |  |  | -.049 [-.062, -.037] | .006 | -.146 | -7.796 | **<.001** |
| Social Support |  |  | .010 [-.002, .021] | .006 | .030 | 1.635 | .102 |
| Religiosity |  |  | .060 [.020, .100] | .020 | .051 | 2.954 | **.003** |
|  | Outpatient: Increase in Burden (*F*(12, 815) = 4.864, *p* < .001, adjusted *R*² = .053) | | | | | | |
|  | *R²* | ∆ *R²* | B (95% CI) | SE | Beta | *t* | *p* |
| Step 1: Control variables | .026 | .026 |  |  |  |  |  |
| Gender^a^ |  |  | .097 [-.081, .275] | .091 | .038 | 1.073 | .284 |
| Age^b^ |  |  |  |  |  |  |  |
| 31-40 |  |  | -.492 [-.840, -.144] | .177 | -.159 | -2.774 | .006 |
| 41-50 |  |  | -.109 [-.469, .251] | .184 | -.040 | -.593 | .553 |
| 51-60 |  |  | -.080 [-.436, .276] | .181 | -.033 | -.442 | .659 |
| >60 |  |  | -.099 [-.495, .297] | .202 | -.031 | -.491 | .624 |
| Professional experience^c^ |  |  |  |  |  |  |  |
| 3-6 years |  |  | .308 [-.257, .873] | .288 | .059 | 1.069 | .285 |
| >6 years |  |  | .648 [.141, 1.155] | .258 | .220 | 2.510 | .012 |
| unknown |  |  | .400 [-.128, .928] | .269 | .108 | 1.487 | .137 |
| Contact with SARS-CoV-2^d^ |  |  | -.023 [-.183, .137] | .081 | -.010 | -.280 | .780 |
| Step 2 | .067 | .040 |  |  |  |  |  |
| Sense of Coherence |  |  | -.066 [-.088, -.044] | .011 | -.225 | -5.870 | **<.001** |
| Social Support |  |  | .017 [-.003, .037] | .010 | .061 | 1.636 | .102 |
| Religiosity |  |  | .008 [-.064, .080] | .037 | .008 | .225 | .822 |

*Notes.* ∆ *R²* = Change in *R².* SE = Standard Error. ^a^ Reference group = male. ^b^ Reference group = Age 18 -30. ^c^ Reference group = Professional experience <3 years. ^d^ Reference group = no.
